# Institutionalisierung der Infektionshygiene in Deutschland: Herausforderungen seit der Gründerzeit: Ein Rückblick auf über 50 Jahre KRINKO

**DOI:** 10.1007/s00103-026-04259-x

**Published:** 2026-06-18

**Authors:** Barbara Buchberger, Jana Maidhof, Esther E. Dirks, Franziska Lexow

**Affiliations:** 1 Robert Koch-Institut, Nordufer 20, 13353 Berlin, Deutschland; 2 Lehrstuhl für Medizinmanagement, Universität Duisburg-Essen, Essen, Deutschland; 3 Abteilung 1 Infektionskrankheiten, Fachgebiet 14 Angewandte Infektions- u. Krankenhaushygiene, Robert Koch-Institut, Berlin, Deutschland

**Keywords:** Infektionshygiene, Robert Koch, KRINKO, Nosokomiale Infektionen, Institutionalisierung, IPC, Infection prevention and control, Robert Koch, KRINKO, Nosocomial infections, Institutionalisation

## Abstract

Die Sicherstellung der öffentlichen Gesundheit durch strukturierte Infektionsprävention ist heute fest verankert. Die Etablierung unabhängiger Institutionen, evidenzbasierter Richtlinien und sektorenübergreifender Kooperationen in der Hygiene war jedoch kein Selbstläufer, sondern Ergebnis tiefgreifender historischer Entwicklungen und gesundheitlicher Krisen. Ziel des Beitrags ist es darzustellen, welche Entwicklungen in Deutschland zur Institutionalisierung der Infektionshygiene geführt haben und welchen wechselnden Herausforderungen sie seit der Gründerzeit begegnen muss.

Durch Industrialisierung und Urbanisierung entstanden im letzten Drittel des 19. Jahrhunderts Probleme in der Wasserversorgung und Abwasserentsorgung, die zu schweren Infektionsausbrüchen führten. Ab den 1920er-Jahren mussten Lösungen für die gesundheitsgefährdende Luft- und Bodenbelastung infolge des technischen Fortschritts entwickelt werden und nach 1950 gerieten nosokomiale Infektionen in den Fokus.

Die Gründung der Königlichen Versuchs- und Prüfungsanstalt für Wasserversorgung und Abwasserbeseitigung 1901 war ein bedeutender Schritt für die Institutionalisierung der Infektionshygiene. Wechselnden Anforderungen entsprechend wurde das Aufgabenspektrum angepasst und zusätzliche Institute gegründet. Durch das Infektionsschutzgesetz sind heute das Robert Koch-Institut und die bei ihm angesiedelte Kommission für Infektionsprävention in medizinischen Einrichtungen und in Einrichtungen der Pflege und Eingliederungshilfe (KRINKO) mandatiert, Aufgaben der Infektionshygiene wahrzunehmen. An Schnittstellen mit anderen für Infektionsprävention und -hygiene zuständigen Einrichtungen des Bundes erzeugt die interinstitutionelle Zusammenarbeit Synergien zum Schutz der Bevölkerung.

## Abstract (en)

The protection of public health through structured infection prevention is now firmly established. However, the establishment of independent institutions, evidence-based recommendations and cross-sectoral cooperation in the field of hygiene was not a foregone conclusion, but rather the result of profound historical developments and health crises. The aim of this article is to describe the developments in Germany that have led to the institutionalisation of infection prevention and control (IPC) and the changing challenges it has faced since its inception.

Industrialisation and urbanisation in the last third of the 19th century led to problems with water supply and sewage disposal, which in turn led to serious outbreaks of infection. From the 1920s onwards, solutions had to be developed for the health hazards posed by air and soil pollution as a result of technological progress, and after 1950, nosocomial infections became the focus of attention.

The establishment of the Royal Research and Testing Institute for Water Supply and Wastewater Disposal in 1901 was a significant step towards the institutionalisation of IPC. The range of tasks was adapted to changing requirements and additional institutes were founded. Under the Infection Protection Act, the Robert Koch Institute and its Commission for Infection Prevention and Hygiene in Healthcare and Nursing are now mandated to perform IPC tasks. At interfaces with other federal institutions responsible for infection control inter-institutional cooperation creates synergies for the protection of the population.

## Einleitung

Die Sicherstellung der öffentlichen Gesundheit durch eine strukturierte Infektionsprävention ist heute eine fest verankerte staatliche und gesellschaftliche Aufgabe. Die Etablierung fachlich unabhängiger Institutionen, evidenzbasierter Richtlinien und sektorenübergreifender Kooperationen im Bereich der Hygiene war jedoch kein Selbstläufer, sondern das Resultat tiefgreifender historischer Entwicklungen und gesundheitlicher Krisen. Ziel der vorliegenden Übersichtsarbeit ist es, den Prozess der Institutionalisierung der Infektionshygiene in Deutschland seit der Gründerzeit nachzuzeichnen und die stetig wechselnden Herausforderungen zu beschreiben, denen sich die staatlichen Institute stellen mussten.

## Folgen des wirtschaftlichen Aufschwungs in Deutschland und Preußen ab 1871

Mit der Reichsgründung 1871 entstanden auch in Deutschland zahlreiche Industriezweige und Großbetriebe zur Rohstoffgewinnung, Eisen- und Metallverarbeitung, Maschinenbau und Chemieproduktion [1]. Der wirtschaftliche Aufschwung der Gründerzeit führte insbesondere in den entstehenden Ballungsräumen in Preußen zu Wasserversorgungsproblemen, denn die Beseitigung flüssiger Abfallstoffe stellte sowohl für die Bevölkerung als auch die Industrie ein Problem dar. Abwasser und Nutzwasser gerieten immer wieder in Kontakt, was zu Ausbrüchen von Infektionskrankheiten wie der Cholera 1892 in Hamburg oder Typhus in Gelsenkirchen 1901 führte [1].

Für die Industrie waren Bergbau und Hüttenwesen als Energielieferanten von immenser Bedeutung und der Bedarf an Arbeitern groß. So konnte beispielsweise im Landkreis Beuthen des oberschlesischen Industriebezirks ein gewaltiger Zuzug von Menschen beobachtet werden: Die Bevölkerung des Dorfes Schwientochlowitz stieg von 9866 im Jahr 1895 auf 18.799 im Jahr 1900 [2]. Zeitgleich boomte der Wohnungsbau und es entstanden lange Straßen mit Häuserfronten von bis zu dreistöckigen Gebäuden, in denen vier bis zehn Familien oder mehr wohnten. Der Baugrund wurde durch zusätzliche Seiten- und Hinterhäuser mit engen Höfen vollständig ausgenutzt. Diese Art der Bebauung mit hoher Bevölkerungsdichte war eine der Ursachen für vermehrt auftretende Epidemien, die Wasserversorgung und Abfallentsorgung wesentliche andere [2].

Der Bekämpfung dieser Epidemien durch das 1876 gegründete Kaiserliche Gesundheitsamt als zentraler Behörde des Medizinal- und Veterinärwesens in Berlin waren die politischen und publizistischen Aktivitäten Salomon Neumanns und Rudolf Virchows ab 1848 vorangegangen. Sie forderten auch im Hinblick auf Seuchenschutz eine soziale Medizin und Bildung für große Bevölkerungsgruppen, die im Übergang von der Agrar- zur Industriegesellschaft verelendet waren [3].

Auf lokaler Ebene gab es im 19. Jahrhundert keine Gesundheitsämter, sondern einzelne Amtsärzte waren im Rahmen der Medizinal- und Sanitätspolizei zuständig; um die Jahrhundertwende entstanden dann ein System von Wohlfahrtseinrichtungen sowie kommunale Gesundheitsämter für die Gesundheitsfürsorge [4]. Zur Spannung zwischen gesundheitspolizeilicher Seuchenabwehr und sozialhygienischer Gesundheitsfürsorge sagte Virchow bereits 1848 nach Entsendung zu einer Fleckfieberepidemie über die Beamten, denen er eine Mitschuld an der Ausbreitung gab: „Waren doch die Beamten nicht von dem Volk für das Volksinteresse, sondern von dem Staat für das Staatsinteresse eingesetzt“ [5].

## Typhusepidemien und Robert Koch

Insbesondere in den Bergbaugebieten in Oberschlesien traten immer wieder Typhusepidemien auf, denn nicht immer waren zentrale Wasserleitungen hygienisch unbedenklich [6, 7]. Obwohl bekannt war, dass Grubenwasser aus aktiven Schächten verunreinigt und nicht für die zentrale Wasserversorgung geeignet war, verursachte Grubenwasser 1897 in der Stadt Beuthen im gleichnamigen Landkreis eine Typhusepidemie, in deren Folge 1618 Menschen erkrankten und 85 starben [2, 8]. Auch Robert Koch verweist am 14.08.1901 in einem Brief auf den Ausbruch in Beuthen. Darin informiert er den Minister der geistlichen, Unterrichts- und Medizinalangelegenheiten eindrücklich über die Bedeutung der Trinkwasserqualität zur Bekämpfung von Typhus sowie über die Krankheitslast für die Gesellschaft:

„Als Beispiel sei daran erinnert, daß während der Typhusepidemie in Beuthen 1897, wo unter 36.000 Einwohnern gegen 2000 an Typhus erkrankten, Verschleppungen der Krankheit bis nach Berlin, Torgau, Karlsbad, Reichenhall beobachtet wurden. Diese verschleppten Fälle aber, welche der Sachlage nach gewöhnlich zuerst gar nicht erkannt werden, bilden deshalb eine so große Gefahr selbst für Ortschaften mit sogenannter guter Wasserversorgung, weil die besten Wasserleitungsanlagen gelegentlich schadhaft werden und das Eindringen von krankmachenden Keimen gestatten. Auf diese Weise ist sogar hier in Berlin vor wenigen Jahren eine recht erhebliche Typhusepidemie entstanden. Es ist sogar auffallend, wie häufig gerade der Typhus epidemisch ausbricht, wenn einmal eine Wasserversorgungsanlage schadhaft wird oder fehlerhaft betrieben wird. Solche Epidemien sind in allerneuester Zeit in Lüneburg, Essen a. d. R., Löbau i. S., Striegau, Sprottau und anderen Orten entstanden.

Diese Epidemien sowie die an die Verseuchung einzelner Brunnen sich anschließenden Typhusfälle weisen mit zwingender Gewalt darauf hin, daß trotz aller Desinfektion noch viel mehr Typhuskeime ausgestreut werden, als es bei oberflächlicher Betrachtung scheinen möchte, und daß wir noch weit davon entfernt sind, für die Unterdrückung des Typhus das zu tun, was jetzt schon in unserer Macht steht. Eine Aufklärung über diese Verhältnisse haben uns erst die letzten Jahre gebracht, wo nachgewiesen wurde, daß die Typhusbakterien nicht nur im Stuhle der Kranken enthalten sind, sondern auch bei einer nicht geringen Anzahl von Typhusfällen in unglaublicher Menge mit dem Urin ausgeschieden werden, und daß selbst nach der völligen Entfieberung des Kranken diese Ausscheidung wochen- und monatelang fortbestehen kann. Dieser Faktor ist bisher von der praktischen Hygiene noch gar nicht gewürdigt worden.

Ähnlich verhält es sich mit den festen Entleerungen der Typhusrekonvaleszenten. Die Frage, wie lange ein von Typhus Genesener noch Typhusbazillen zu entleeren vermag, kann noch nicht mit genügender Sicherheit zahlenmäßig beantwortet werden. Daß es sich aber nicht selten um Wochen und Monate handelt, ist durch gewissenhafte Beobachtungen festgestellt. Dann kommen für uns noch die leichten Fälle in Betracht, welche gar nicht für Typhus gelten, aber ebensoviel Typhusbazillen ausstreuen und deshalb ebenso gefährlich sind wie diese“ [9].

## Die Gründung der Königlichen Versuchs- und Prüfungsanstalt für Wasserversorgung und Abwasserbeseitigung 1901

Urbanisierung und Industrialisierung erforderten den Bau von Anlagen zur zentralen Trinkwasserversorgung und Abwasserentsorgung. In hygienischer und technischer Hinsicht waren sie jedoch oft mangelhaft, weil Sachverständige mit hydrogeologischen Kenntnissen für die Beratung der Kommunalverwaltungen und der Industrie fehlten [1, 8]. Die von den Hygienikern seit Längerem geforderte Grundwassergewinnung zur Wasserversorgung konnte sich daher gegenüber dem genutzten Oberflächenwasser mit potenzieller Gesundheitsgefährdung nicht durchsetzen; zusätzlich fehlten Verfahren zu einer adäquaten Aufbereitung von Abwasser [1]. Auch ein persönliches Interesse von Industriellen und privaten Sachverständigen war oft nicht auszuschließen [10]. Ein Erlass des preußischen Ministeriums vom 24.08.1899, in dem für neue Anlagen zur Wasserversorgung eine hygienische Prüfung und sanitätspolizeiliche Beobachtung angeordnet wurden, konnte den Missstand mangels qualifizierter Fachleute auch nicht beheben [1].

Die Gefährdung der Bevölkerungsgesundheit durch Epidemien und damit einhergehende volkswirtschaftliche Verluste erhöhten den Druck auf die Medizinalverwaltungen, mit der Planung eines separaten und neutralen Instituts zu beginnen. Dessen Mitarbeiter sollten Evidenz zur Wasserhygiene generieren, diese kritisch beurteilen und in Empfehlungen für die Praxis umsetzen [8, 10]. Auch die Wirtschaftlichkeit der Anlagen zur Wasserversorgung und Abwasserbeseitigung sollte durch das Institut gewährleistet werden [10]. Je nach Interesse zwischen einer eher wissenschaftlichen Ausrichtung vonseiten der technischen Hochschulen und Universitäten und einer praxisnahen Ausrichtung, die der Industrie und den Kommunen ein Anliegen war, variierten jedoch die Meinungen über die Organisation und Anbindung der Einrichtung [1, 8].

Am 01.04.1901 kam es schließlich zur Gründung der Königlichen Versuchs- und Prüfungsanstalt für Wasserversorgung und Abwasserbeseitigung mit Sitz in Berlin, Kochstraße 73 [1]. Um interessengeleitete Forschung zu vermeiden, wurden die Mitarbeiter verbeamtet [8, 10]. Zu Beginn unterstand die Anstalt dem Preußischen Minister der geistlichen, Unterrichts- und Medizinalangelegenheiten. In diesem Ministerium war der Obermedizinalrat Adolf Schmidtmann beschäftigt, der erkannt hatte, dass gesetzgeberische Maßnahmen allein nicht ausreichten, sondern der Staat auch Verantwortung für Forschung auf dem Gebiet zu übernehmen hatte. Insbesondere die fehlende Grundlagenforschung hatte die Entwicklung von Standards verhindert und die Unsicherheit bei den nachgeordneten Behörden und Medizinalverwaltungen erhöht [1]. Vor dem preußischen Abgeordnetenhaus erklärte Schmidtmann diese Ausrichtung: „Die Anstalt ist und soll kein wissenschaftliches Institut der hergebrachten Art sein, sondern ein wissenschaftliches Institut, welches vor allen Dingen praktischen Zielen und Zwecken dient“ [1, 8].

Die Aufgaben umfassten praxisnahe Forschung zu Wasserverhältnissen, Verunreinigung und Verseuchung von Gewässern, Überwachung der Wasserqualität und Beratung von Kommunen und Industrie. Darüber hinaus war das Institut für die Aus- und Weiterbildung von Beamten in der Gewerbeaufsicht und Medizin sowie Sanitätsoffiziere und Beschäftigte im Brunnenbau und in der Schädlingsbekämpfung zuständig [8, 10].

Um Brücken zwischen den verschiedenen technischen und naturwissenschaftlichen Disziplinen des Umwelt- und Gesundheitsschutzes sowie den unterschiedlichen Praxisakteuren zu schlagen, gründeten Vertreter der Kommunen und der Industrie in Abstimmung mit dem Ministerium am 28.02.1902 den Verein für Wasserversorgung und Abwasserbeseitigung [11]. Zweck des Vereins war es, die Königliche Versuchs- und Prüfungsanstalt für Wasserversorgung und Abwasserbeseitigung bei ihren Aufgaben durch Mitwirkung und Fördergelder zu unterstützen [8]. Gerade die finanziellen Zuwendungen waren für die Anstalt wichtig, weil es so möglich wurde, junge Wissenschaftler für Forschungsprojekte erst als Angestellte des Vereins zu beschäftigen, bevor sie nach ihrer Bewährung in der Anstalt verbeamtet wurden [8]. Der Verein wurde im Jahr 1952 umbenannt in „Verein für Wasser‑, Boden- und Lufthygiene“ (WaBoLu). Er zählt zu den ältesten Fördervereinen im Bereich der Umwelt- und Siedlungshygiene [12].

## Der Typhusausbruch in Gelsenkirchen

Exemplarisch für das Ringen der Disziplinen und Interessenvertreter um eine adäquate Gesundheitsversorgung war der Typhusausbruch in Gelsenkirchen im Jahr 1901, in dessen Folge 3231 Menschen erkrankten und 350 starben [1]. Um die zwei Direktoren, den Ingenieur und den Maschinenmeister der städtischen Wasserwerke, zur Rechenschaft zu ziehen, wurde im Jahr 1904 ein Prozess eingeleitet, der auf der Grundlage des ersten Lebensmittelgesetzes in Deutschland von 1879 aufbaute [13, 14]. Unklar war, ob dem Trinkwasser absichtlich Abwasser aus der Ruhr beigemischt worden oder ein Rohrbruch aufgetreten war, der zur Verbreitung des Typhuserregers geführt hatte.

Zu den geladenen Sachverständigen gehörten neben Rudolf Emmerich, einem Schüler Max von Pettenkofers, Robert Koch sowie Arthur Springfeld, ein weiterer preußischer Medizinalbeamter. Letzterer ging von einem Unfall aus, was auch Koch nach seiner Entsendung durch das Ministerium 1901 in einem öffentlichen Vortrag als Ausbruchsursache angegeben hatte [14]. Vor Gericht widerrief er diese Aussage, die er bereits am 21.10.1901 auch in einem Schreiben an den Minister der geistlichen, Unterrichts- und Medizinalangelegenheiten präzisiert hatte, und erklärte sie damit, dass die öffentliche Stimmung gegen die Wasserwerke so erhitzt gewesen sei, dass er sie nicht zusätzlich befeuern wollte; seine Glaubwürdigkeit vor Gericht hatte damit allerdings Schaden genommen [14, 15].

Im Verlauf der Verhandlung wurde erörtert, ob Wasser überhaupt als Gegenstand des Lebensmittelgesetzes verstanden werden könne, und Emmerich betonte, dass Wasser zwar lebensnotwendig sei, aber das sei auch Luft, und die könne sicher nicht als Lebensmittel gelten. Darüber hinaus vertrat er vehement Pettenkofers Miasmen-Theorie über aus dem Boden aufsteigende, krankmachende Dämpfe, und die Verteidiger schlossen ihr Plädoyer mit der Aussage ab, dass Pettenkofers Theorie nicht veraltet sei und man einer einzigen Autorität nicht blind vertrauen dürfe, das habe ja auch der Tuberkulin-Skandal um Koch gezeigt [14].

**Abb. 1:**
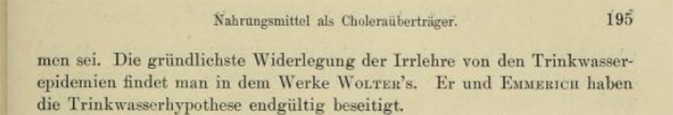
Über Trinkwasserepidemien [16]

Die vier Angeklagten wurden zwar verurteilt, aber der Fall ist bemerkenswert, weil im Jahr 1904 eigentlich niemand mehr Zweifel am Übertragungsweg von Typhus und Cholera haben konnte. Gegner von Robert Koch wie Friedrich Wolter und Rudolf Emmerich fanden jedoch lange Gehör und noch 1912 schrieb Georg Sticker in seinem Standardwerk über die Cholera, dass die Lehre von den Trinkwasserepidemien falsch sei (Abb. 1; [16]).

Für den Großraum Gelsenkirchen regte Koch nach der Typhusepidemie die Gründung einer Institution zur Hygieneüberwachung an und bereits im Dezember 1901 wurde der „Verein zur Bekämpfung der Volkskrankheiten im Ruhrkohlengebiet“ gegründet. Der Verein baute ein Hygiene-Institut auf und verfolgt bis heute das Ziel, wissenschaftliche Projekte zu unterstützen, die mit dem Fokus auf Umwelthygiene und Toxikologie dem Gesundheitsschutz oder dem Schutz der Umwelt dienen. Darüber hinaus bietet er Fortbildungen durch das Personal des Instituts an [17].

Im Frühjahr 1902 initiierte Robert Koch die systematische Typhusbekämpfung im Südwesten Deutschlands unter der Leitung von Paul Frosch. In Trier und Saarbrücken wurden Labore für bakteriologische Untersuchungen aufgebaut und in der Folgezeit als höchst relevante Einrichtungen der Infektionshygiene verstetigt [18].

## Entwicklung der Anstalt, Neubau des Institutsgebäudes und Umbenennung

**Abb. 2:**
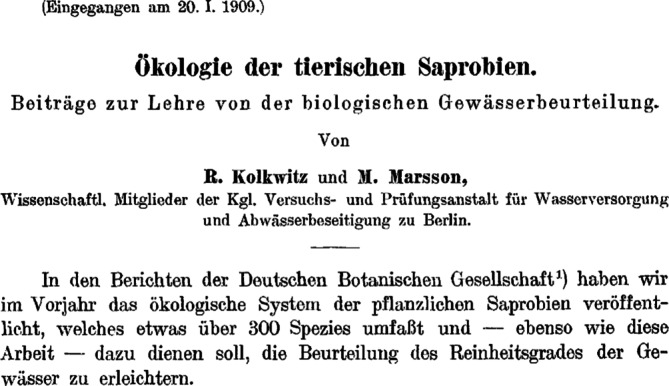
Publikation zur Beurteilung von Wasserqualität aus der Königlichen Versuchs- und Prüfungsanstalt für Wasserversorgung und Abwässerbeseitigung zu Berlin [19]

Von anfänglich zehn stieg die Anzahl der Mitarbeiter der Königlichen Versuchs- und Prüfungsanstalt für Wasserversorgung und Abwasserbeseitigung stetig an. Dem Verständnis für die Bedeutung der wissenschaftlichen Arbeit entsprach eine großzügige Zuweisung von Mitteln [1]. Zu den ersten maßgeblichen Publikationen aus der Anstalt zählen 1904 die „Grundsätze für Anlage und Betrieb von Grund- und Wasserwerken“ sowie 1908 die „Ökologie der tierischen Saprobien“ durch die Hydrobiologen Richard Kolkwitz und Maximilian Marsson zur Beurteilung von Wasserqualität (Abb. 2; [19]) – im Gedenken an die Leistung von Richard Kolkwitz stiftete der Verein WaBoLu 80 Jahre nach seiner Gründung die Richard-Kolkwitz-Plakette, die Persönlichkeiten verliehen wird, die sich verdienstvoll der Umwelthygiene gewidmet haben [1, 11].

**Abb. 3:**
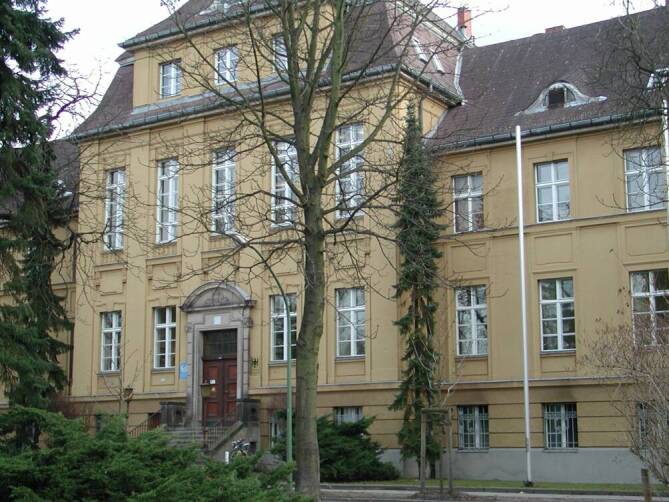
Königliche Versuchs- und Prüfungsanstalt für Wasserversorgung und Abwasserbeseitigung (Quelle: Umweltbundesamt)

Welche Bedeutung der Anstalt zugemessen wurde, spiegelt der ab 1911 am Corrensplatz in Berlin Dahlem entstehende Kolossalbau mit einer 100 m langen Schaufront wider, in den die Mitarbeiter 1913 einzogen. Heute ist er eine Liegenschaft des Umweltbundesamts (UBA; Abb. 3).

Nach dem Ersten Weltkrieg entstanden neue technische, wissenschaftliche und wirtschaftliche Herausforderungen für den Gesundheitsschutz. Infolge der weiter zunehmenden Industrialisierung mussten Lösungen für die Entsorgung fester Abfallstoffe wie Müll, Schutt und Kadaver gefunden werden und auch die Luftverschmutzung durch Staub und Abgase wurde zum Problem [1]. Wie bei der Ab- und Trinkwasserhygiene 30 Jahre zuvor fehlte es auch in den 1920er-Jahren an wissenschaftlichen Grundlagen und technischen Möglichkeiten, auf deren Basis Behörden geeignete Maßnahmen hätten ergreifen können [1]. Die Regierung reagierte mit der Erweiterung des Aufgabenspektrums und einer Umbenennung des Instituts im April 1923 in „Preußische Landesanstalt für Wasser‑, Boden- und Lufthygiene“ [1, 8].

Dass die Landesanstalt für Wasser‑, Boden- und Lufthygiene den gewaltigen Sparmaßnahmen der preußischen Regierung infolge der Inflation nicht zum Opfer fiel, war dem Verein für Wasser‑, Boden- und Lufthygiene zu verdanken. Die Reputation und das entschlossene Auftreten der Mitglieder führten bei einer Sitzung im Ministerium für Volkswohlfahrt 1924 dazu, dass die Anstalt ohne jegliche Einschränkungen erhalten blieb [1]. Zwar hatte die drohende Auflösung damit abgewendet werden können, aber die Anzahl der Aufträge für Gutachten für Behörden, Kommunen und Industriebtriebe stieg in einem solchen Ausmaß an, dass die von der Regierung nicht bewilligten zusätzlichen Planstellen die wissenschaftliche Arbeit der Anstalt bedrohten. Wieder musste der Verein für Wasser‑, Boden- und Lufthygiene einspringen, um dem Personalnotstand abzuhelfen [1]. Im Vergleich zum Stand vor dem Ersten Weltkrieg hatten sich die Mitgliederzahl und damit die Beitragsgelder des Vereins nahezu verdoppelt. Er war in der Lage, die Personalkosten für zahlreiche junge Wissenschaftler zu bezuschussen, die unter Anleitung und bei geringer Bezahlung Lösungen für drängende Probleme technischer und naturwissenschaftlicher Art erarbeiten konnten; gleichzeitig wurde Fachpersonal ausgebildet, mit dem Planstellen in der Zukunft besetzt werden konnten [1].

## Entwicklung im Nationalsozialismus und nach dem Zweiten Weltkrieg

Als für Seuchenbekämpfung zuständige Einrichtungen wurden das Preußische Institut für Infektionskrankheiten „Robert Koch“ und die Preußische Landesanstalt für Wasser‑, Boden- und Lufthygiene 1935 dem Reichsgesundheitsamt unterstellt [20]. Dort wurde 1936 auch eine Rassenhygienische Forschungsstelle eingerichtet, die wie der öffentliche Gesundheitsdienst auf der Grundlage des Gesetzes über die Vereinheitlichung des Gesundheitswesens (GVG) von 1934 agierte, dem zentralen Instrument zur Erfassung und Selektion im Dienst der nationalsozialistischen Erbpflege und Rassenhygiene [21].

Während des Zweiten Weltkrieges war das in Reichsanstalt für Wasser- und Luftgüte umbenannte Institut überwiegend mit der Überwachung des Brauch- und Abwassers der Rüstungsindustrie betraut und wurde mit zunehmender Materialknappheit gutachterlich für die Freigabe von Baustoffen zum Neubau von Wasser- und Abwasseranlagen tätig [1]. Infolge des Luftkriegs wurde die Anstalt auch für den Schutz der Wasserwerke gegen Bombenschäden und die Sicherstellung einer hygienisch unbedenklichen Trinkwasserversorgung der Bevölkerung verantwortlich; Forschungstätigkeiten wurden im Lauf des Krieges immer stärker eingeschränkt [1].

Nach dem Krieg wurde das wenige verbliebene oder zurückgekehrte Personal der Anstalt zur Überwachung der stark zerstörten zentralen Wasserversorgung Berlins, der einzelnen Brunnen, der Abwasseranlagen und zur Schädlingsbekämpfung eingesetzt [1]. Seuchen konnten auf diese Weise verhindert werden und auch die Wasserversorgung und Abwasserentsorgung wurden auf einem gewissen Sicherheitsniveau möglich [1]. Unmittelbar nach Kriegsende hatte der Magistrat von Groß-Berlin das Robert Koch-Institut, die Landesanstalt für Wasser‑, Boden- und Lufthygiene und Teile des Personals aus dem ehemaligen Reichsgesundheitsamt als dem Infektionsschutz dienende Institute aufgrund aufgehobener gesetzlicher Grundlagen in der Arbeitsgruppe B der Abteilung Gesundheitswesen zusammengefasst [1, 8, 22]. Aus dieser Arbeitsgruppe ging am 23.10.1945 das „Zentralinstitut für Hygiene und Gesundheitsdienst“ hervor und 1948 durch Neugestaltung das Robert Koch-Institut für Hygiene und Infektionskrankheiten [1, 8, 22].

Nach Gründung der Bundesrepublik Deutschland erging am 27.02.1952 der Erlass des Gesetzes über die Einrichtung eines Bundesgesundheitsamtes [1, 8]. Als Basis dienten die beiden bereits vorhandenen Institute, das Robert Koch-Institut zur Erforschung und Bekämpfung von Infektionskrankheiten und das Institut für Wasser‑, Boden- und Lufthygiene für den gesundheitlichen Umweltschutz; ein Institut für Hygiene und Toxikologie, Ernährungsmedizin, Zoonosen und bakterielle Veterinärmedizin wurde eingerichtet, dem der Name Max von Pettenkofers verliehen wurde [22]. In den 1970er-Jahren traten neben einer Zentralabteilung für Verwaltung vier weitere Institute dazu: das Institut für Sozialmedizin und Epidemiologie, das von einer Abteilung zu einem Institut aufgewertete Institut für Strahlenhygiene, das Institut für Veterinärmedizin (Robert von Ostertag-Institut) und das Institut für Arzneimittel – 1989 wurde zusätzlich das AIDS-Zentrum als Ausgliederung einer Abteilung des Robert Koch-Instituts eingerichtet [22, 23]. Dem Institut für Wasser‑, Boden- und Lufthygiene war 1957 nach Integration der entsprechenden Labore aus dem Max von Pettenkofer-Institut die Zusatzbezeichnung „Forschungsstätte für allgemeine Hygiene und Gesundheitstechnik“ verliehen worden, da das Aufgabenspektrum um Fragen der Heizung, Lüftung, Beleuchtung und Klimatechnik erweitert worden war [8].

## Die Gründung der Kommission Hygiene und Gesundheitstechnik in Operations- und Spezialpflegebereichen 1972

Der Aus- und Neubau von Krankenhäusern sowie die Etablierung von Intensivstationen in den 1950er-Jahren erfolgten unter dem Eindruck von Epidemien wie der großen Polioepidemie in Skandinavien 1952. Diese hatte dazu geführt, dass 1954 die weltweit erste Intensivstation in Dänemark eröffnet wurde [24]. In Deutschland entstand die erste Intensivstation 1957 am Westend-Krankenhaus der Freien Universität Berlin mit dem expliziten Ziel der Beatmung von Poliopatienten und als Reanimationszentrum [24]. Ungefähr zeitgleich entwickelten sich Intensivstationen in den USA aus den Überwachungsstationen für Patienten mit koronarer Herzkrankheit [24].

Im Verlauf der Zeit wurden die zunehmenden, zum Teil schweren und auch letalen Sekundärinfektionen, der sogenannte Hospitalismus, und steigende Antibiotikaresistenzen weltweit zu einem Problem. In Deutschland führte die Situation zu einem Anstieg der Beratungsersuchen der Länder beim Bundesgesundheitsamt. Wie auch in der Zeit von Robert Koch wurde der Mangel an Fachleuten beklagt [25, 26].

**Abb. 4:**
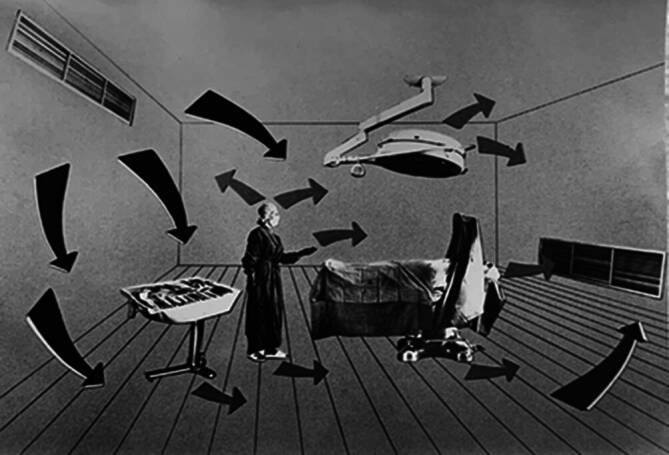
Problematik der Aseptik in Operationsräumen. Primäre Luftkeime (*schwarze Pfeile*), sekundäre Luftkeime (*graue*, *kleine Pfeile*). (Vortrag anlässlich der Mitgliederversammlung der Forschungsvereinigung für Luft- und Trocknungstechnik E. V. am 15.06.1972 „Problematik der Aseptik in Operationsräumen“ Prof. Dr. med. Kanz, München; Schriftstück im RKI vorhanden)

**Abb. 5:**
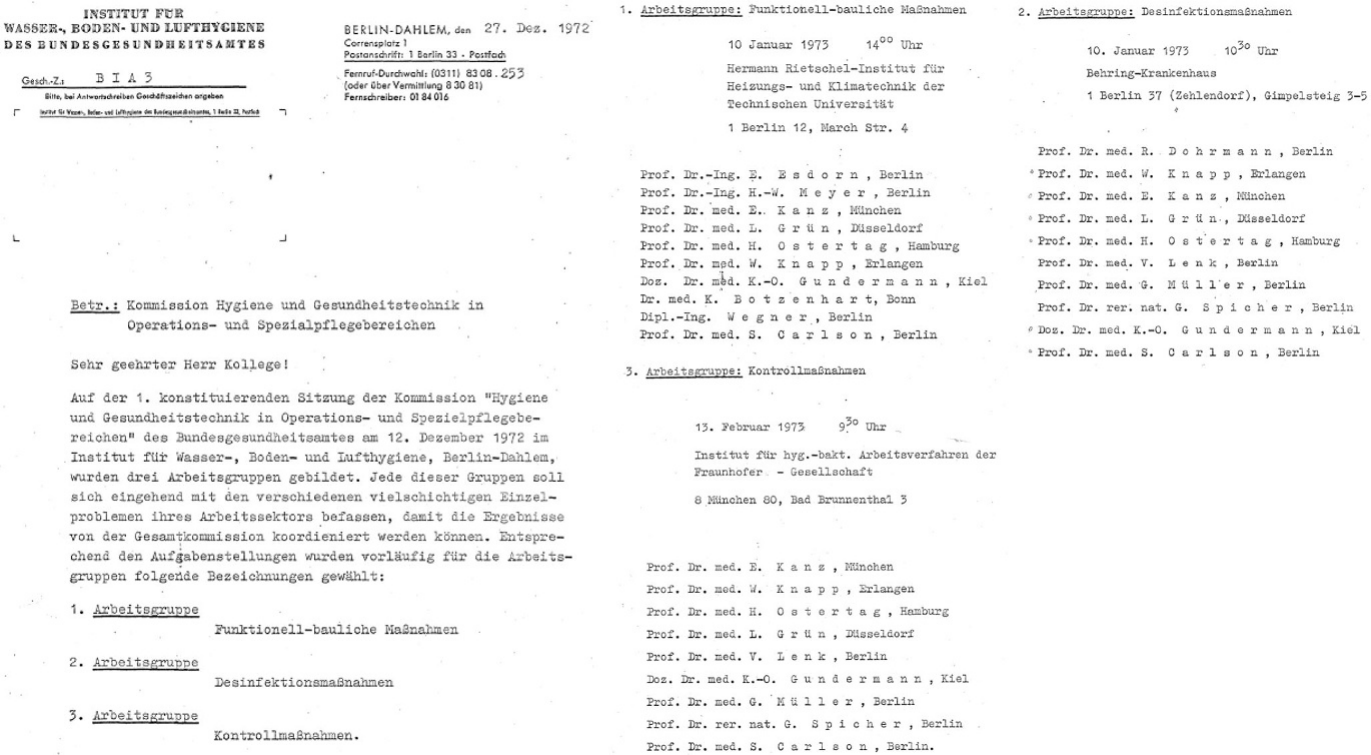
Schreiben des ersten Direktors und Professors Dr. med. Friedrich Höffken aus dem Institut für Wasser‑, Boden- und Lufthygiene des Bundesgesundheitsamtes und die ersten 3 Arbeitsgruppen der Kommission Hygiene und Gesundheitstechnik in Operations- und Spezialpflegebereichen (Quelle: RKI)

Akute Fälle von Gasbrandinfektionen durch Clostridien bei Operationen in Hamburg (Abb. 4) brachten das Bundesgesundheitsamt und dessen Direktor Georg Henneberg ins Handeln: Aus dem Institut für Wasser‑, Boden- und Lufthygiene erging am 27.12.1972 durch den Leiter Friedrich Höffken ein Schreiben mit dem Bericht über die konstituierende Sitzung der Kommission „Hygiene und Gesundheitstechnik in Operations- und Spezialpflegebereichen“ (Abb. 5; [27]). Es wurden 3 Arbeitsgruppen gebildet, die sich im Januar und Februar 1973 in Berlin und München trafen.

## Anpassung des Aufgabenspektrums und Umbenennungen der Kommission

Zwei Jahre nach der konstituierenden Sitzung erfolgte eine Umbenennung in „Kommission für Krankenhaushygiene“ und nach intensiver Arbeit konnte 1976 die umfassende und grundlegende „Richtlinie zur Erkennung, Verhütung und Bekämpfung von Krankenhausinfektionen“ veröffentlicht werden [27, 28].

Diese Richtlinie wurde unter Berücksichtigung medizinischer Entwicklungen wie der Einführung technisch-komplexer Medizinprodukte (z. B. Endoskope) oder infektionsepidemiologischen Herausforderungen u. a. durch die Verbreitung des HI-Virus (HIV) über die Jahre fortgeschrieben. Dem Gedanken, Infektionshygiene auch wieder über Krankenhäuser hinausgehend zu betrachten, entsprach 1989 die Umbenennung in „Kommission für Krankenhaushygiene und Infektionsprävention“ (KRINKO). Infolge des Skandals um mit HIV kontaminierte Blutprodukte wurde 1994 das Bundesgesundheitsamt aufgelöst [29]. Auch die Arbeit der KRINKO wurde unterbrochen, bis die Kommission 1997 wiederberufen wurde [27].

Ausgehend von der Arbeitsgruppe um David Sackett an der kanadischen McMaster University verbreitete sich die moderne evidenzbasierte Medizin auch in Europa [30] und die KRINKO reagierte 1999 mit einer Reform ihrer Arbeitsweise durch die Einführung von Evidenzkategorien.

Die Bedeutung der Prävention von nosokomialen Infektionen und Infektionen durch multiresistente Erreger erkannte auch der Gesetzgeber und erließ im Jahr 2001 das Gesetz zur Verhütung und Bekämpfung von Infektionskrankheiten beim Menschen, das Infektionsschutzgesetz (IfSG). Darin sind die Aufgaben der KRINKO beschrieben, insbesondere der Auftrag, evidenzbasierte Empfehlungen zur Infektionsprävention sowie zu betrieblich-organisatorischen und baulich-funktionellen Maßnahmen der Hygiene in Krankenhäusern und anderen medizinischen Einrichtungen zu erstellen.

Als sich Ende des 20. Jahrhunderts nicht nur in Nordamerika und Australien, sondern auch in Deutschland multiresistente Erreger wie Methicillin-resistenter *Staphylococcus aureus* (MRSA) massiv ausbreiteten [31], reagierte die KRINKO mit der Entwicklung erregerspezifischer sowie spezieller Empfehlungen zum Ausbruchsmanagement bei gehäuftem Auftreten nosokomialer Infektionen [32]. Infolge der COVID-19-Pandemie wurde im Jahr 2022 das IfSG angepasst sowie die KRINKO in „Kommission für Infektionsprävention in medizinischen Einrichtungen und in Einrichtungen und Unternehmen der Pflege und Eingliederungshilfe“ umbenannt. Gemäß § 23 IfSG erstellt die Kommission nunmehr nicht nur Empfehlungen zur Prävention von nosokomialen, sondern auch von weiteren Infektionen. Darüber hinaus müssen Einrichtungen und Unternehmen der Pflege und Eingliederungshilfe nach § 35 IfSG nun auch im Rahmen der Durchführung medizinischer oder pflegerischer Maßnahmen die Empfehlungen der KRINKO berücksichtigen (Infobox).

## Fazit

Wechselnden Herausforderungen dynamisch zu begegnen, kann seit den Anfängen der Institutionalisierung von Infektionshygiene als ihr Motto gelten. Die hier skizzierte Entwicklung mit dem Fokus auf staatliche Einrichtungen war jedoch weder konfliktfrei noch geradlinig und auch die fast vollständige Durchdringung der Institute mit der nationalsozialistischen Ideologie muss betont werden.

Hohen Erwartungen ist auch die KRINKO nach über 50 Jahren ausgesetzt: Die ehrenamtlich arbeitende Kommission soll die bestmögliche Evidenz für infektionspräventive Maßnahmen zusammentragen, die in sehr heterogenen Settings praktikabel und umsetzbar sein sowie unter Berücksichtigung neuer infektionsepidemiologischer Erkenntnisse stetig weiterentwickelt werden müssen. Da die Arbeit der KRINKO der Verbesserung von Prozessen in medizinischen und pflegerischen Einrichtungen dient, ergeben sich im Bereich der Belüftung oder Wasserentsorgung Überschneidungen mit anderen Instituten und Behörden wie dem UBA oder dem Bundesinstitut für Risikobewertung (BfR). Für den Öffentlichen Gesundheitsdienst organisieren das RKI, UBA und BfR daher einmal im Jahr eine gemeinsame Fachtagung.

Mit der Einbeziehung von Nachhaltigkeitsaspekten unter Aufrechterhaltung optimaler infektionshygienischer Standards für Patienten und Personal oder aber auch durch die Beschäftigung mit fundamentalen Themen wie der Wasserhygiene in medizinischen Einrichtungen passt sich die KRINKO der Dynamik von wechselnden Herausforderungen für die Hygiene an und knüpft in gewisser Hinsicht, allerdings unter gänzlich anderen Voraussetzungen, wieder an die Probleme der Medizinalverwaltungen zur Zeit Robert Kochs an. Wie damals stellen der Fachkräftemangel und die sowohl interdisziplinäre als auch intersektorale Zusammenarbeit die größten Herausforderungen für die Kommission dar.

### Infobox Fakten zur KRINKO

**Zusammensetzung**

➢ Geschäftsstelle beim RKI

➢ Unabhängige, ehrenamtlich tätige Kommission

➢ I. d. R. alle drei Jahre vom Bundesministerium für Gesundheit berufen

➢ Ca. 20 Mitglieder unterschiedlicher Fachdisziplinen (z. B. Krankenhaushygiene, Chirurgie, ÖGD)

**Arbeitsweise**

➢ Arbeitsplan: Bildung von Arbeitsgruppen → Empfehlungsentwurf → Beratung in der KRINKO → Öffentliches Stellungnahmeverfahren durch Länder und Fachgesellschaften → Beratung der Rückmeldungen → Veröffentlichung RKI

➢ Empfehlungen mit Evidenzkategorien

➢ Modulare Struktur

➢ Übersetzung relevanter KRINKO-Dokumente ins Englische

**Herausforderungen**

➢ *Evidenzsynthese:* Nicht für jede medizinische und pflegerische Maßnahme liegen epidemiologische Untersuchungen zum Auftreten von NI vor, da sie zum Teil aus ethischen Gründen nicht durchführbar sind.

➢ *Arbeitspensum:*

   • Überprüfung bestehender Empfehlungen auf Aktualität und Evidenz
   • Entwicklung neuer Empfehlungen aufgrund neuer relevanter Krankheitserreger (z. B. Candidozyma auris) bzw. medizinisch-technischer Innovationen
   • Viele parallel arbeitende Arbeitsgruppen

➢ *Heterogener Adressatenkreis: *z. B. Arztpraxen, Universitätskliniken, Pflegedienste, Rettungsdienste, Eingliederungshilfe, ÖGD

➢ *Praktikabilität:* Verhältnismäßigkeit zwischen bestmöglichem Infektionsschutz und Gewährleistung der Patientenversorgung

➢ *Kommunikation:* Komplexe Sachverhalte verständlich darstellen und zugänglich machen

*KRINKO* Kommission für Infektionsprävention in medizinischen Einrichtungen und in Einrichtungen und Unternehmen der Pflege und Eingliederungshilfe; *RKI* Robert Koch-Institut; *ÖGD* Öffentlicher Gesundheitsdienst.

## Data Availability

Alle dieser Arbeit zugrunde liegenden Daten sind in diesem Artikel enthalten.
